# Supplementary material for: Echogenic intracardiac foci detection and location in the second-trimester ultrasound and association with fetal outcomes: A systematic literature review
Source: PLoS One. 2024 Apr 22;19(4):e0298365. doi: 10.1371/journal.pone.0298365 (PMC11034667; doi:10.1371/journal.pone.0298365)
Supplement: S1 Table — (DOCX) [file pone.0298365.s001.docx]

S1: Extracted variables, Definitions and How They Were Recorded

| **Category Name** | **Explanation** | **Data options** |
| --- | --- | --- |
| **Study Context** |  |  |
| First author | Surname | Text |
| Year | Year of publication | Date |
| Country | The country of origin that the data used in the study is taken from | Text |
| Healthcare setting | This is the source of Electronic Health Record data used in the analysis | Text |
| Prospective or retrospective | e.g. retrospective looking at medical records, or, prospectively scanning/assessing baby | Text |
| **Data** |  |  |
| Total number of foetuses | Sample size | Number |
| Follow up period | Birth, post-natal | Number |
| Number and prevalence of EIF | per 100 | Number |
| Number of foetuses without EIF | Per 100 | Number |
| Prevalence of preterm among EIF | Per 100 | Number |
| Prevalence of preterm among those without EIF | Per 100 | Number |
| Cardiac abnormality recorded | Y N | Text |
| Detail of cardiac abnormality |  | Text |
| Cardiac abnormality recorded non EIF | Per 100 | Number |
| Detail of cardiac abnormality non EIF | Per 100 | Number |
| Chromosomal abnormality recorded | Per 100 | Number |
| Details of chromosomal abnormality | Text | Text |
| Multiple EIF recorded | 0- No 1-Yes |  |
| Detail of Multiple EIF | Text field | Text |
| Left, right, bilateral |  | Text |
| Details of Left, Right, Bilateral |  | Text |
| Termination of pregnancy (TOP)/death recorded | Number of deaths | Number |
| Details of TOP/death | Text | Text |
| Additional details and limitations noted. | Text | Text |
| **CASP** |  |  |
| Is the question focused? | Y N |  |
| Was cohort recruited in an acceptable way? | Y N |  |
| Was exposure (EIF) accurately measured? | Y N |  |
| Was outcome measured appropriately? | Y N |  |
| Have confounding factors been identified (e.g. excluded those with known abnormalities)? | Y N |  |
| Was the period of follow up sufficient? | Y N |  |
| Quality score (out of 6) | Number of Test scores | Number |
| Comments on quality |  | Text |
