# Supplementary material for: Echogenic intracardiac foci detection and location in the second-trimester ultrasound and association with fetal outcomes: A systematic literature review
Source: PLoS One. 2024 Apr 22;19(4):e0298365. doi: 10.1371/journal.pone.0298365 (PMC11034667; doi:10.1371/journal.pone.0298365)
Supplement: S2 Table — (DOCX) [file pone.0298365.s002.docx]

S2: Specific findings from included studies which examine cardiac outcomes.

| Alipour et al (2022) | N=114 women referred for foetal echocardiography in Iran were examined. N=7 were referred due to EIF and there was 1 case (14%) with a cardiac abnormality [1]. |
| --- | --- |
| Huang et al (2021) | Follow-up of N=192 women with EIF in China who were undergoing amniocentesis. Of these, N=75 (39%) also had cardiac malformation such as VSD with left ventricular EIF, left ventricular EIF and tricuspid regurgitation or left EIF and right ventricular EIF and mitral regurgitation, and tricuspid regurgitation [34]. |
| Song et al (2021) | N=150 babies with EIF examined in China. EIFs identified in early or mid-trimester did not carry a risk but persistence of EIF in late trimester was associated with heart defects after the birth. 82 with a single EIF (9 (10%) which developed congenital heart disease) and 68 with multiple EIF (5 (7%) with CHD) suggesting no increased risk of congenital heart disease if in those with multiple EIF compared to single EIF. Of those with CHD this included VSD, and valve defect, EIF remained after the birth in 2 cases [26]. |
| Ladak et al (2021) | Follow-up of N=1909 women having a foetal echocardiogram in Pakistan. 168 (28%) had EIF. 3 (1.8%) had major congenital health defects and they concluded that a referral for EIF alone was not warranted for low-risk cases, especially in low resource settings [3]. |
| Ozsurmeli et al (2020) | Follow-up of N=233 foetuses with EIF in Turkey. 8 (3%) showed cardiac abnormalities [37]. |
| Akinmoladun et al (2020) | Follow-up of N=44 babies with EIF in Nigeria. 42 with 1 focus and 2 with multiple, 2 had the EIF within the right ventricle and 1 had on both left and right. Of the 44 babies, 5 (25%) had structural cardiac anomalies (tricuspid regurgitation, VSD, accelerated flow in the aorta, hypoplastic left heart) and of these 3 had major structural anomalies, these were all infants with the EIF in the left ventricle [15]. |
| Chiu et al (2019) | Follow-up of N=531 babies with EIF in China. 455 were in the left and 18 had multiple foci, of these 9 (2%) had structural defects (including 5 VSD) and 11% of EIFs remained until the third trimester. 29 in the right, 3 multiple foci and of these 1 (3.4%) had structural defects (VSD) and 34% of these remained until the third trimester. 47 with both sided intracardiac EIF and of these 2 (4.2%) had structural defects (VSD, double outlet right ventricle) and 13% remained until third trimester. The positive predictive value of an EIF for cardiac defects was 2.26% [38]. |
| Guo et al (2018) | Follow-up of N=2647 babies with EIF in China. N=2,498 were on the left and of these 93 (3.7%) had cardiac abnormality. There were 45 with right sided EIF and of these 5 (11.1%) had cardiac abnormality. 104 with bilateral, of whom 3 (2.9%) had cardiac abnormality. The most common cardiac abnormalities were VSD, tetralogy of Fallot and pulmonary stenosis. The rate of cardiac abnormality was 2.3% for those with multiple EIFs and this was not a higher rate than those with single EIF. The study concluded that foetuses with right sided EIF tend to present with cardiac anomalies more frequently [14]. |
| Shakoor et al (2013) | Follow-up of N=71 babies in Pakistan with EIF. 2 had multiple EIFs. 46 were in the left, 3 in the right and 9 bilaterally. The follow-up in this study was not complete and only women who opted for a scan were followed up. There were 3 (4.2%) identified cases of cardiac abnormality (VSD, one Tetralogy of Fallot, and one Pulmonary artery hypertension) [39]. |
| Lu et al (2017) | Follow-up of N=343 babies in China with EIF. 1 had a heart deformity. There were 3 stillbirths where it was not clear if these were related to heart defects [27]. |
