# Supplementary material for: Echogenic intracardiac foci detection and location in the second-trimester ultrasound and association with fetal outcomes: A systematic literature review
Source: PLoS One. 2024 Apr 22;19(4):e0298365. doi: 10.1371/journal.pone.0298365 (PMC11034667; doi:10.1371/journal.pone.0298365)
Supplement: Attachment — Submitted filename: Response Letter.docx [file pone.0298365.s004.docx]

**Response Letter**

Many thanks for the opportunity to respond to and address the concerns on the manuscript PONE-D-23-31742. A concern was raised about the literature review only including articles up to 2022. To clarify, the literature review was carried out in June 2023. However no relevant papers in 2023 were identified in the literature search therefore the most recent article included in the review is from 2022.

A second concern was raised about a lack of sufficient information on the methodology. This has been addressed and more information on the methodology has been added to meet the recommendations set out by PRISMA. We have mentioned which databases were used for the literature search and have attached a PRISMA checklist to the supplementary information.

Thank you for the comments and feedback and we hope we have addressed these points accordingly.

I hope you consider this manuscript.

Many thanks

Hope Jones
