# Supplementary material for: Echogenic intracardiac foci detection and location in the second-trimester ultrasound and association with fetal outcomes: A systematic literature review
Source: PLoS One. 2024 Apr 22;19(4):e0298365. doi: 10.1371/journal.pone.0298365 (PMC11034667; doi:10.1371/journal.pone.0298365)
Supplement: Attachment — Submitted filename: Response to Reviewers.docx [file pone.0298365.s005.docx]

**Response to Reviewers**

1. Please ensure that your manuscript meets PLOS ONE's style requirements, including those for file naming. The PLOS ONE style templates can be found at

https://journals.plos.org/plosone/s/file?id=wjVg/PLOSOne_formatting_sample_main_body.pdf and

https://journals.plos.org/plosone/s/file?id=ba62/PLOSOne_formatting_sample_title_authors_affiliations.pdf

**Amendments have been made to meet the requirements.**

3. Please include a copy of Table 2 which you refer to in your text on page 13.

**Thank you for this comment, this table is not presented in this manuscript, so this has been removed**

4. Please review your reference list to ensure that it is complete and correct. If you have cited papers that have been retracted, please include the rationale for doing so in the manuscript text, or remove these references and replace them with relevant current references. Any changes to the reference list should be mentioned in the rebuttal letter that accompanies your revised manuscript. If you need to cite a retracted article, indicate the article’s retracted status in the References list and also include a citation and full reference for the retraction notice.

**The reference list has been updated and now includes 2 new recently published articles.**

**Reviewer #1:** This is an exciting study, as the authors have assembled data from past published papers using the Critical Appraisal Skills Programme methodology. The material is generally well written and structured. This review has tried to establish that echogenic intracardiac foci detection and location alone are not associated with adverse outcomes for infants. I especially think that the inclusion and exclusion criteria were very strict but appropriate. The left-sided EIFs are not predictive of poor fetal outcomes, as this information is beneficial for pregnancy and facilitates appropriate clinical guidelines for appropriate medical intervention.

**Reviewer #2:** “Echogenic intracardiac foci detection and location in the second-trimester ultrasound and association with fetal outcomes: A systematic literature review “well drafted and transparent literature review is to examine the association between EIF and infant outcomes.

Minor points:

To the manuscript, I suggest including the study PMID: 32000445. I would like to suggest that authors compare the yield of chromosomal abnormalities if they consider that it is irrelevant.

**We apologise, we had included this reference, but this was not clear as we had not listed the reference to the 10 included studies in the text, we have now added the references to the text to make this clear:**

**Results section: “Ten studies [22, 35, 28, 19, 36, 37, 38, 18, 32, 39] reported chromosomal abnormalities”**

**PMID 32000445 is reference 37 in this list or He et al, 2020.**

1. More fetal echocardiographic characteristics (such as LV, RV, CO, CCO, CCI, SV, FS, etc.) should be included in the beginning, in my opinion.

**Thank you very much for this suggestion, we have examined the echocardiographic characteristics of Left ventricle and Right ventricle, multiple and singular. However, there were not enough publications that examined cardiac output (CO), combined cardiac output (CCO), cardiac index (CI), combined cardiac index (CCI), stroke volume (SV) or fractional shortening (FS). They are available in one recent study published after our review has finished - Zhang et al 2023 [1]. This study looked at 59 women with hypothyroidism and found there was cardiac remodeling in fetuses with a mother with hypothyroidism and EIF was detected more often in those with hypothyroidism. However, the article did not report if the remodeling was associated with those with EIF. This study is on a highly selected population (women with hypothyroidism) and so we have not included it in our review, as we can not detect outcome by EIF status.**

1. **Yanhong Zhang, Lisi Zhang, Wei Zhao, Na Li, Guihong Chen, Jun Ge, Xingna Su, Shuping Ge & Congxin Sun (2023) Cardiac structural and functional remodeling in the fetuses associated with maternal hypothyroidism during pregnancy, The Journal of Maternal-Fetal & Neonatal Medicine, 36:1, DOI: 10.1080/14767058.2023.2203796**

**We have now updated our discussion to include recent findings from papers published after the dates of our review. The following has been added to the discussion:**

**That there is a link between EIF and cardiac abnormality is supported by Taksande et al [54] who recently published a systematic review and meta-analysis and found a prevalence of 4.8% among studies published between 1987-2021. This is comparable to our finding which included 7 of the same studies (but not a meta-analysis) from 2013-2023. The Taksande [54] analysis did not look at left vs right EIF and did not compare with those without EIF. Our findings suggest, when compared to those without EIF in the same study, only a right sided EIF carries significantly higher risk of cardiac abnormality. A follow-up study by Hurt et al [55] published 2023, which followed up the infants with EIF and without EIF for 10 years found no evidence of association of EIF with cardiac admissions or diagnosis of cardiac abnormalities but did find an increased risk of respiratory admissions (Hazard ratio 1.27 (95% CI: 1.04-1.54). The Hurt study did not look at left vs right EIF but the association with respiratory conditions may support the hypothesis that right sided EIF carries morbidity risk more than left sided EIF.**

2. Page 4: Add the title “Introduction “

**This has been added**
